# Development and Validation of Ecofriendly HPLC-MS Method for Quantitative Assay of Amoxicillin, Dicloxacillin, and Their Official Impurity in Pure and Dosage Forms

**DOI:** 10.1155/2021/5570938

**Published:** 2021-06-17

**Authors:** Atiah H. Almalki, Essraa A. Hussein, Ibrahim A. Naguib, Eglal A. Abdelaleem, Hala E. Zaazaa, Fatma F. Abdallah

**Affiliations:** ^1^Department of Pharmaceutical Chemistry, College of Pharmacy, Taif University, P.O. Box 11099, Taif 21944, Saudi Arabia; ^2^Addiction and Neuroscience Research Unit, College of Pharmacy, Taif University, Al-Hawiah, Taif 21944, Saudi Arabia; ^3^Pharmaceutical Analytical Chemistry Department, Beni-Suef University, Alshaheed Shehata Ahmad Hegazy St., Beni-Suef 62514, Egypt; ^4^Analytical Chemistry Department, Pharmacy-Cairo University, Kasr El-Aini, Cairo 11562, Egypt

## Abstract

Novel, accurate, selective, and rapid high-performance liquid chromatography mass spectrometry method was developed for simultaneous analysis of amoxicillin trihydrate, dicloxacillin sodium, and their official impurity 6-aminopenicillanic acid. The chromatographic separation was carried out by applying the mixture on a C_18_ column (3.5 *µ*m ps, 100 mm × 4.6 mm id) using acetonitrile:water (65 : 35 by volume) as a mobile phase within only 4 min. The quantitative analysis was executed using single quadrupole mass spectrometer in which electrospray ionization, selected ion monitoring, and negative mode were operated. The retention times were 1.61, 2.54, and 3.50 mins for amoxicillin, 6-aminopenicillanic acid, and dicloxacillin, respectively. The method was validated in linear ranges of 2–28 *µ*g mL^−1^, 2–35 *µ*g mL^−1^, and 1–10 *µ*g mL^−1^ for amoxicillin, dicloxacillin, and 6-aminopenicillanic acid, respectively. The results obtained from the suggested HPLC/MS were statistically compared with those obtained from the reported HPLC method, where no significant difference appeared respecting accuracy and precision. According to the analytical eco-scale assessment method, the proposed method was proved to be greener than the reported one, where the analysis time and the amount of the wasted effluent decreased.

## 1. Introduction

**Figure 1:**
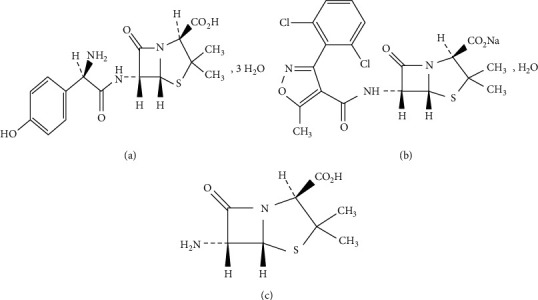
The chemical structure of AMOX (a), DIC (b) and their common impurity 6-APA (c).

Amoxicillin trihydrate (AMOX; Figure 1(a)) [1] and dicloxacillin sodium (DIC; Figure 1(b)) [1] are classified as *β*-lactam antibiotics. Chemical names of AMOX and DIC are 2*S*, 5*R*, 6*R*)-6-[[(2*R*)-2-amino-2-(4-hydroxyphenyl)acetyl]amino]-3, 3-dimethyl-7-oxo-4-thia-1-azabicyclo[3.2.0]heptane-2-carboxylic acid trihydrate and monosodium (2*S*, 5*R*, 6*R*)-6-[[[3-(2, 6-dichlorophenyl)-5-methylisoxazol-4-yl]carbonyl]amino]-3, 3-dimethyl-7-oxo-4-thia-1-azabicyclo[3.2.0]heptane-2-carboxylate, respectively [1]. The pharmacological action of these antibiotics depends on preventing bacterial cell wall synthesis. DIC resists penicillin-resistant bacteria in which *β*-lactamase enzymes played a critical role. [2]. 6-Aminopenicillanic acid (6-APA), (2*S*, 5*R*, 6*R*)-6-amino-3, 3-dimethyl-7-oxo-4-thia-1-azabicyclo[3.2.0]heptane-2-carboxylic acid (Figure 1(c)), is, as of yet, known to be the common contaminant for both AMOX and DIC [3]. This problem usually referred to the fact that 6-APA is the common active nucleus, essential for the synthesis of semisynthetic penicillin antibiotics in pharmaceutical industries. Although 6-APA has been regarded mainly as the starting block material for a wide range of synthetic penicillins, it shows some antibacterial activity as well. 6-APA has been reported to have less activity against Gram-positive bacteria when compared with penicillin G, whereas its activity is relatively slightly better against Gram-negative organisms [4].

Many efforts have been devoted to the quantification of the two studied active components either in the authentic form or in the commercial pharmaceutical form. In fact, there are numerous methods that were reported for amoxicillin determination, such as spectrophotometry [5–9], voltammetry [10, 11], fluorimetry [12, 13], HPLC [14, 15], and HPLC in biological fluids [16–18]. Similarly, DIC has been determined using spectrophotometry [19–22], HPLC [23–26], and HPTLC [27]. More intriguingly, AMOX and DIC were detected in their binary mixture using spectrophotometric methods [28–32] and chromatographic methods [33–35]. Ternary mixture of DIC, ampicillin, and 6-APA was analyzed by HPTLC [36]. Additionally, DIC, AMOX, and many antibacterial agents were analyzed in poultry feathers by UPLC method [37] However, the reported methods offered lower efficiency towards the separation of AMOX and DIC in the presence of 6-APA either in pure form or in the formulation. Naguib et al. [38] developed UV spectrophotometry coupled with chemometric methods for the simultaneous determination of AMOX, DIC, and 6-APA; however, the use of support vector machines SVR is well known to be computationally intense and requires special codes and software, which makes it not friendly for quality control purposes.

HPLC has been acquainted as a robust separation technique with high selectivity. Most of the reported HPLC methods showed poor ability to differentiate between closely related chemical compounds and high consumption of mobile phase, relative to the rarely reported HPLC methods coupled with a mass spectrometer (HPLC/MS), which offer, on the other hand, rational consumption of the mobile phase and higher selectivity and sensitivity. Accordingly, the objective of this study was to establish and validate an HPLC/MS method, as a powerful method offering high sensitivity and selectivity, for the analysis of a mixture of DIC, AMOX, and 6-APA. The benefits anticipated from this work are to combine the advantages of the chromatographic methods, such as the effective separation and selective determination of each component in the mixture, together with the advantages of attaching the mass detection where more accurate detection, as well as high sensitivity for the drugs of interest, is granted. In addition to HPLC/MS merits, the analysis time of the presented method is very low, that is, less than 4 mins, which offers rational consumption of the mobile phase. Eco-scale quantitative assessment [39] was additionally applied to compare the developed method to the reported method [33] with respect to the environmental aspects [39], where the proposed HPLC/MS was found to be greener than the reported one [33].

The presented HPLC/MS method does not need sophisticated software like support vector regression SVR used by previously developed chemometric methods [38], which make it friendlier to quality control analysts.

## 2. Materials and Methods

### 2.1. Apparatus

The HPLC instrument was (Agilent 1260 Infinity, Germany) connected with a preparative pump (G1361A). The HPLC was also connected to quadrupole LC/MS detector (Agilent 6120) and diode array detector VL (Agilent 1260 Infinity, G131SD). In addition, the HPLC was also equipped with thermostated column compartment (Agilent 1260 Infinity, G1316A) and preparative Autosampler (Agilent 1260 Infinity, G2260A). The quantitation and separation were processed on Eclipse Plus C_18_ column (3.5 *µ*m particle size, 100 mm × 4.6 mm i.d) (USA).

- Xz6 bench top laboratory centrifuge with maximum speed to 5000 rpm and time from zero to sixty minutes
- Sonix TV series ultrasonicator (USA)

### 2.2. Material and Reagents

#### 2.2.1. Pure Standard

Pure forms of the drugs of interest and 6-APA impurity were imported through Sigma-Aldrich, the Egyptian International importing and exporting trade Center (EIC, Egypt).

#### 2.2.2. Pharmaceutical Dosage Form

Miclox^®^ was as capsules formula produced by MISR Co. For Phar. Ind. S.A.E. Each capsule was claimed to contain 500 mg of AMOX and DIC with ratio (1 : 1); 250 mg of each drug.

#### 2.2.3. Solvents and Chemicals

- Methanol (E.Merck, Germany), HPLC grade
- Acetonitrile (Sigma-Aldrich, Germany), HPLC grade
- Water for injection according to B.P. 2003 was from (Egypt Otuska Pharmaceutical Co., S.A.E., 10^th^ of Ramadan city, A.R.E)

#### 2.2.4. Standard Solutions

The standard solutions were prepared as

- The first stock standard solutions of all studied components were prepared in pure methanol as 1000 *µ*g mL^−1^.
- The corresponding standard working solutions of all investigated components were prepared after accurate dilutions by methanol to reach a concentration of 100 *µ*g mL^−1^ for the respective component. All standard solutions were freshly made and kept in the refrigerator to be used for the HPLC analysis within 24 h.

## 3. Experimental

### 3.1. HPLC-MS Conditions

Chromatographic separation implemented isocratic approach on C_18_ (3.5 *µ*m ps, 100 mm *x* 4.6 id) column. The mobile phase made from acetonitrile:water (65 : 35, vol/vol) was conducted at a flow rate of 0.5 mL min^−1^ and temperature of 25°C. The injected volume was controlled to be 20 *µ*L. The LC running time was just 4 minutes. Quadrupole mass spectrometer was used with electrospray ionization (ESI) at negative mode for molecules deprotonation to detect the mass of analyzed drugs. Selected ion monitoring (SIM) mode was operated to record only specific m/z values [40, 41] (74.1, 223.0 and 443.9 for 6-APA, AMOX, and DIC respectively). Capillary voltage was at 3000 V, and drying gas temperature was at 350°C.

### 3.2. Linearity and Construction of Calibration Curves

Series concentrations of (100 *µ*g mL^−1^) were prepared by transferring an accurately measured aliquot equivalent to 20–280 *µ*g of AMOX, 20–350 *µ*g of DIC, and 10–100 *µ*g of 6-APA, into 10-mL volumetric flasks, and volume was made up with the mobile phase. Although the mobile phase is running, the three components were separated and detected by a mass detector according to their molecular ions. After that, the calibration curve and regression equations were calculated using the relative mass areas (drug mass area/mass area of a constant concentration of the drug).

### 3.3. Assessment of Pharmaceutical Dosage Form

The ingredient of 20 capsules was assembled, weighed, and blended well. Then, 100 mg of AMOX and DIC, which were equivalently weighed, was moved into 100-mL volumetric flask, and 75 mL of purified water was added. After that, 1 mg mL^−1^ stock solution was obtained by sonication of a mixture of the solution for 30 min, cooling, and completing to volume with purified water. The resulted solution was then filtered and diluted to get a 100 *µ*g mL^−1^ working solution. Procedures mentioned in linearity and constructions of calibration curves were applied to get concentrations of AMOX and DIC from their related regression equations.

## 4. Results

6-APA, which is the core chemical structure of all penicillins, was found to have less efficacy when compared with penicillin G for the treatment of Gram-positive bacteria, whereas against Gram-negative organisms, the discrepancy in activity is relatively low [4]. The work in this article introduces a highly selective chromatographic method (HPLC/MS) that is able to analyze both studied antibiotics quantitatively in the presence of their reported common impurity for the first time, 6-APA in pure form and in their binary pharmaceutical mixture, offering high selectivity and sensitivity.

### 4.1. HPLC/MS Results

**Figure 2:**
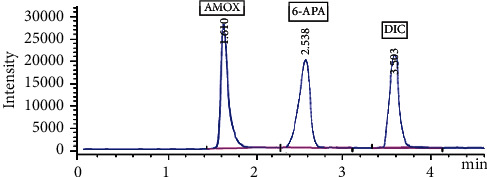
HPLC/MS chromatogram of resolved mixture of 20 *µ*g mL^−1^ of AMOX, 10 *µ*g mL^−1^ of 6-APA, and 20 *µ*g mL^−1^ of DIC (*R*_*t*_ = 1.610, 2.538, and 3.503 min, respectively) using (acetonitrile:water (65 : 35 by volume) as mobile phase.

HPLC/MS method was developed to be sensitive, accurate, and highly selective for the quantitative determination of the studied components following ICH guidelines [38], which recommend very specific conditions for levels of impurities in pharmaceutical products. The separation process was conducted where acetonitrile:water (65 : 35, vol/vol) was implemented as a mobile phase, and the flow rate was at 0.5 mL min^−1^. The retention times were 1.61, 2.54, 3.50 min for AMOX, 6-APA, and DIC, respectively, as shown in Figure 2. Using LC-MS method allows the analysis of compounds of interest at early times without fears of interfering solvent peaks, where mass detection offers necessary high selectivity compared with UV detector, and low analysis time allows the consumption of less mobile phase, which allows the method to be greener.

The calibration curves that were made for the three components were created by plotting the relative mass area (drug mass area/mass area of a constant concentration of the drug) against the corresponding concentration.

The regression equations were determined as follows:

*Y* = 0.1027 *X* + 0.1656, *r* = 0.9997 for AMOX.

*Y* = 0.0999 *X* + 0.0042, *r* = 0.9998 for DIC.

*Y* = 0.4803 *X* + 0.0388, *r* = 0.9997 for 6-APA.where *Y* is the relative mass area, *X* is the concentration in *µ*g mL^−1^, and *r* is the correlation coefficient, as shown in Table 1.

## 5. Discussion

### 5.1. Method Optimization

#### 5.1.1. Chromatographic Conditions

*(1) Mobile Phase Composition*. The mobile phase composition was optimized to achieve good resolution and sharp peaks with a short analysis time. Several aqueous phases were tried, such as water acidified with formic acid and water with triethylamine; however, purified water is the best aqueous phase partner that achieves acceptable system suitability parameters and allows the detection with mass spectrometer. Regarding the organic phase, methanol and acetonitrile were tried and acetonitrile showed better resolution, especially between AMOX and 6-APA. Increasing the acetonitrile ratio enhances peak shape and symmetry. Finally, acetonitrile/water (65 : 35 vol/vol) system could achieve the purpose of resolution successfully.

*(2) Flow Rate*. Several flow rates were tried, including 0.4, 0.5, 0.8, and 1 mL min^−1^; however, best resolution could be achieved using 0.5 mL min^−1^, yet providing very short analysis time.

#### 5.1.2. Mass Spectrometry

**Figure 3:**
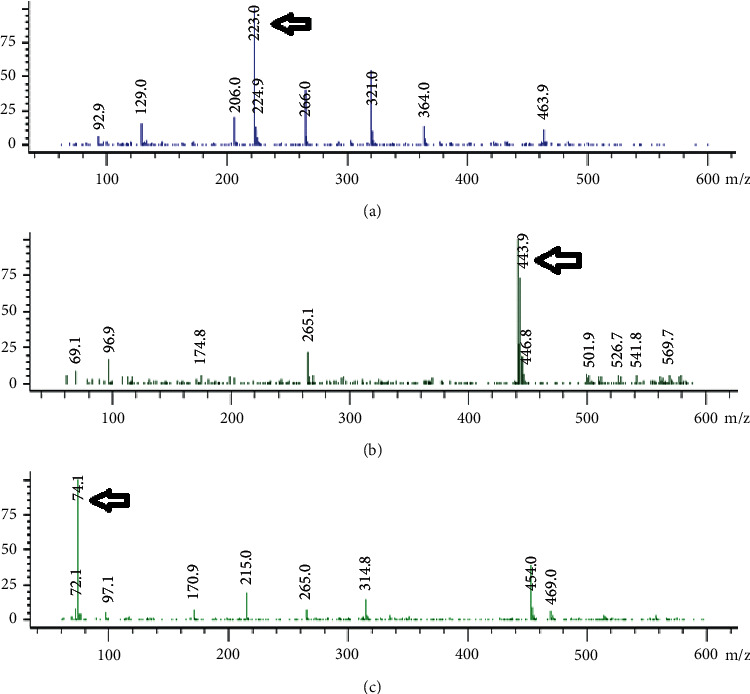
The mass spectra for the three components with their *m*/*z* (74.1, 223.0 and 443.9 for 6-APA (c), AMOX (a), and DIC (b), respectively).

Many factors were optimized to attain the highest response for AMOX, DIC, and 6-APA simultaneously. The suitable ion mode was detected by testing the positive and negative ion modes. Only DIC was detected in positive and negative ion modes, whereas for AMOX and 6-APA, the negative ion mode response was found to be much more sensitive than the positive ion mode. The three components were determined with high sensitivity and selectivity with the negative ion mode. In this work, a single quadrupole mass spectrometer was used with “scan” mode first and then “SIM” mode. From the scan mode, full-mass spectrum was obtained for each component, which relates the m/z with abundance.  Figure 3 shows the mass spectra of AMOX, DIC, and 6-APA, showing the ion of interest at *m*/*z* = 223.0, 443.9, and 74.1, respectively [40, 41]. In SIM mode, the instrument was set to monitor only specific *m*/*z* values (74.1, 223.0, and 443.9 for 6-APA, AMOX, and DIC, respectively). After the components were separated by the chromatographic mobile phase, they were detected specifically by mass detector according to *m*/*z* values as in Figure 2.

### 5.2. Application to the Pharmaceutical Formulation

The suggested HPLC/MS method was effectively employed for assay of AMOX and DIC in capsules form [Miclox® (0.5 gm)], displaying good percentage recoveries. Further assessment was conducted using the standard addition technique to validate the proposed method, as shown in Table 2.

The results mentioned above presented an accurate and highly selective method for the quantitative analysis of AMOX and DIC in the existence of 6-APA. The HPLC/MS method showed high sensitivity because it has the ability to determine the reported impurity even in concentrations far below the concentration of the drug itself. Hence, the HPLC/MS method is considered to be a valuable tool for testing the purity and quantity of drug product.

### 5.3. Quantitative Assessment of the Greenness of the Developed HPLC-MS Method

One of the commonly used green metrics is the analytical eco-scale, which is a semiquantitative assessment tool to estimate the greenness of a given analytical method [39]. Various parameters and steps were compared by this tool for the whole analytical process.

Any factor employed in the analytical procedure is expressed by evaluative penalty points, which are used to construct the analytical eco-scale of the studied analytical method. Ideal green analytical method has eco-scale total score of 100. If the total eco-scale score exceeds 75, the method is assessed as excellent, whereas methods with a total eco-scale score exceeding 50 are acceptable. Analytical method is expressed as an inadequate green analytical method if the total eco-scale score is below 50 [39, 42].

Penalty points are specified for each of the four main parameters of a given analytical procedure (hazardousness, energy consumption, amount of reagents, and waste production) that represents the ideal green analytical method. For reagents, penalty points are given specific hazard categories—environmental, physical, and health—that each reagent presents. Varying amounts of a given reagent will show varying penalty points. Penalty points for hazards are relying on the “Globally Harmonized System of Classification and Labeling of Chemicals (GHS)” [39]. Each analytical reagent has one or more of nine pictograms, which are a graphic representation of inherent hazardous properties. Two signal words are used in GHS: “danger” (i.e., more severe hazard and equal to 2 penalty points) and “warning” (i.e., less severe hazard and equal to 1 penalty point). Other points are specified relying on the used instruments, consumed energy, and type and amount of waste [39, 42].

The correlation regarding the analytical eco-scale between the proposed HPLC-MS method and the reported HPLC method was established (Table 3). The proposed HPLC method proved to be greener than the reported method, where the overall analytical eco-scale score was (75) for the proposed method and (71) for the reported method (Table 3). It is worthily to note that the amount of effluent and analysis time is much less than the reported one.

## 6. Method Validation

Validation of the introduced method was executed in correspondence with the international conference on harmonization (ICH) guidelines [43].

### 6.1. Linearity

All components of interest were analyzed by HPLC/MS method in triplicates, and the resulted values were linear in the range of 2–28 *µ*g mL^−1^, 2–35 *µ*g mL^−1^, and 1–10 *µ*g mL^−1^, respectively, Table 1.

### 6.2. Accuracy

Accuracy of the proposed method was evaluated through implementation of the standard addition technique and analyzing market pharmaceutical formulations by the proposed HPLC/MS method. Good recoveries of the added pure forms affirmed good accuracy of the suggested method (Table 2).

### 6.3. Precision

#### 6.3.1. Repeatability

Three concentrations of AMOX (4, 12, and 24 *µ*g mL^−1^), DIC (5, 15, and 25 *µ*g mL^−1^), and 6-APA (2, 6, and 10 *µ*g mL^−1^) were analyzed three times to estimate the intraday variation on the same day. Good values of RSD% were listed in Table 1.

#### 6.3.2. Intermediate Precision

The same concentrations were assessed by the aforementioned procedures repeatedly seven times during four successive days to confirm the intermediate precision of the presented method. Good values of RSD % are listed in Table 1.

### 6.4. Specificity

The proposed methods demonstrated specificity by good separation of AMOX, DIC, and 6-APA. Good separation was evaluated by different retention times (1.610, 2.538, and 3.503 min for AMOX, DIC, and 6-APA, respectively), Figure 2.

### 6.5. Detection and Quantitation Limits (LOD and LOQ)

Quantitation and detection limits were calculated mathematically [43]. Acceptable detection and quantitation limits are listed in Table 1.

LOD = 3.3 × SD/slope LOQ = 10 × SD/slope [43].

### 6.6. Robustness

The robustness of an analytical procedure was used to provide an indication of its reliability during normal usage by measuring its ability to endure unaffected by small deliberate deviations in method parameters [43].

For HPLC/MS method, robustness was calculated by making little intentional small variations in the chromatographic conditions. The relative standard deviations (%RSD) are calculated, indexed, and given in Table 4.

### 6.7. System Suitability

System suitability tests were defined according to ICH as an integral part of many analytical methods, particularly liquid chromatographic approaches. These tests were usually conducted to confirm that both reproducibility and resolution of the developed procedure were appropriate to the analysis. System suitability parameters are calculated, as shown in Table 5.

The results that were attained using the suggested HPLC/MS were statistically compared with those provided by the reported HPLC method [33]. It had been shown that no significant difference was displayed when comparing the values of the proposed method with that of the reported one with respect to accuracy and precision as calculated using Student-t test and F-values (Table 6).

The benefits of the presented work are combining the advantages of the chromatographic methods, such as the effective separation and selective determination of each component in the mixture, together with the advantages of attaching the mass detection where more accurate detection, as well as high sensitivity for the drugs of interest, is granted. In addition to HPLC/MS merits, the analysis time of the presented method is very low, that is, less than 4 mins, which offers rational consumption of the mobile phase, and, accordingly, a more environmentally friendly method. Additionally, the used mobile phase is greener compared with the previously published HPLC method [33] that was used for comparison, which uses 1% CH_3_COOH acid solution:acetonitrile: (53 : 47, vol/vol) as mobile phase (Table 3). The presented HPLC/MS method does not need sophisticated software like support vector regression SVR used by previously developed chemometric methods [38], which makes it friendlier to quality control analysts.

## 7. Conclusion

The achieving targets of the presented research work can be concluded in separation and simultaneous quantitation of the ternary mixture of AMOX, DIC, and 6-APA and constructing a comparative study regarding the penalty points between the newly presented HPLC/MS method and the previously published one relying on analytical eco-scale semiquantitative assessment method. According to the analytical eco-scale total values, the presented method is greener than the reported method.

The presented HPLC/MS method is validated with respect to ICH guidelines and proved to be highly selective, accurate, and reproducible. The method also provides high specificity and the ability to determine the reported impurity at very low concentration levels, unlike the reported chemometric method that was unable to quantitate 6-APA. HPLC/MS is a powerful technique that combines the advantage of HPLC separation with the specificity of mass detection in a short analysis time (4 min).

Based on the foregoing, the proposed HPLC/MS method can be applied in quality control and routine analysis of the studied drugs without interference of usually contained pharmaceutical preparation additives.

## Figures and Tables

**Table 1:** Validation analytical parameters of the developed HPLC/MS method for the simultaneous assessment of AMOX, DIC, and 6-APA.

Parameter	AMOX	DIC	6-APA
Calibration range (*µ*g mL^−1^)	2–28	2–35	1–10
Slope	0.1027	0.0999	0.4803
Intercept	0.1656	0.0042	0.0388
Mean %	99.67%	99.97%	100.20%
SD	1.751	1.285	1.145
Correlation coefficient (*r*)	0.9997	0.9998	0.9997
LOD^*∗*^	0.436	0.539	0.232
LOQ^*∗*^	1.322	1.634	0.702
RSD%^a^^*∗∗*^	0.132–0.885 – 0.463	0.533–0.186–0.764	0.787–0.238 – 0.519
RSD%^b^^*∗∗*^	1.341–1.702 – 0.945	1.556–0.820–1.174	1.540–1.306 – 1.169

^*∗*^Limit of detection and quantitation are mathematically calculated (LOD = 3.3 × SD of the response/slope, LOQ = 10 × SD of the response/slope). ^*∗∗*^(RSD%)^a^^*∗∗*^ and (RSD%)^b^^*∗∗*^, the intra- and interday relative standard deviations of concentrations (4, 12, and 24 *µ*g mL^−1^) for AMOX, (5, 15, and 25 *µ*g mL^−1^) for DIC, and (2, 6, and 10 *µ*g mL^−1^) for 6-APA.

**Table 2:** Determination of AMOX and DIC in Miclox® capsules (Batch no. 001011) by the proposed HPLC/MS method and application of standard addition technique.

AMOX				DIC			
Taken (*µ*g mL^−1^)	Found %^*∗*^ ± SD	Pure added (*µ*g mL^−1^)	Recovery %	Taken (*µ*g mL^−1^)	Found %^*∗*^ ± SD	Pure added (*µ*g mL^−1^)	Recovery %
10.00	99.63 ± 1.237	6.00	101.14	10.00	102.65 ± 1.297	5.00	97.94
		10.00	99.30			10.00	100.75
		12.00	102.55			15.00	97.43

Mean **±** SD			**100.99** **±** **1.632**	Mean **±** SD			**98.71** **±** **1.788**

^*∗*^Average of 6 determinations.

**Table 3:** Penalty points of the reported HPLC method and the proposed HPLC-MS method.

Reagent/Instrument	Penalty points	
	Reported HPLC [33]	Proposed HPLC-MS
Acetonitrile	20	20
Acetic acid	4	0
Energy	0	2
Waste	5	3
Occupational hazards	0	0
Total penalty points	Σ 29	Σ 25
Analytical eco-scale total score	71	75

**Table 4:** Experimental results of robustness for the determination of AMOX and DIC with their common impurity, 6-APA, by the proposed HPLC/MS method.

Parameters (%RSD)	AMOX	DIC	6-APA
Acetonitrile/water (60: 40, vol/vol)	1.034	1.121	1.188
Acetonitrile/water (70: 30, vol/vol)	1.019	1.377	1.458
Flow rate (0.4 mL/min)	0.900	0.785	0.290
Flow rate (0.8 mL/min)	1.092	1.579	1.440

**Table 5:** Parameters of system suitability of the developed HPLC method for the determination of AMOX and DIC with their common impurity, 6-APA.

Parameters	AMOX	6-APA	DIC	Reference value [43]
Resolution (*R*_*s*_)	1.427 1.544			>1.5
Selectivity (*α*)	1.576 1.380			>1
Capacity factor (*K*′)	0.610	1.538	2.503	1–10
Symmetry factor	0.916	0.889	1	*T* = 1 for a typical symmetric peak
Number of theoretical plates (*N*)	115.204	210.333	785.345	Increases with efficiency of the separation
HETP (cm/plate)	0.086	0.047	0.012	The smaller the value, the higher the column efficiency

**Table 6:** Statistical analysis of the proposed HPLC/MS method and the reported HPLC method for the determination of AMOX and DIC in pharmaceutical formulations.

Parameters	AMOX, HPLC/MS	DIC, HPLC/MS	Reported HPLC method [33] ^*∗∗*^	
			AMOX	DIC
Mean	99.63	102.65	100.03	102.80
SD	1.237	1.297	1.568	1.222
Variance	1.530	1.683	2.460	1.493
N	6	6	6	6
Student's *t*-test^*∗*^**(2.228)**	0.633	0.835	ـــــ	ـــــ
*F*-test^*∗*^**(5.050)**	1.608	1.127	ـــــ	ــــــ

^*∗*^The values between parenthesis are corresponding to the theoretical values of *t* and *F* (*P*=0.05). ^*∗∗*^ HPLC determination of AMOX in binary mixture with DIC using acetonitrile: 1% acetic acid solution (47 : 53, vol/vol) as mobile phase and UV detection at 240 nm [33].

## Data Availability

Data that support results can be sent upon request.
